# A semi-automated spectral approach to analyzing cyclical growth patterns using fish scales

**DOI:** 10.1093/biomethods/bpae018

**Published:** 2024-03-18

**Authors:** Julien A Chaput, Gérald Chaput

**Affiliations:** Earth, Environmental, and Resource Sciences, University of Texas at El Paso, El Paso, TX 79968, United States; Science Branch, Fisheries and Oceans Canada, Moncton E1C 9B6, Canada

**Keywords:** semi-automated, fast Fourier transform, image analysis, fish scales

## Abstract

We introduce a new semi-automated approach to analyzing growth patterns recorded on fish scales. After manually specifying the center of the scale, the algorithm radially unwraps the scale patterns along a series of transects from the center to the edge of the scale. A sliding window Fourier transform is used to produce a spectrogram for each sampled transect of the scale image. The maximum frequency over all sampled transects of the average spectrogram yields a well-discriminated peak frequency trace that can then serve as a growth template for that fish. The spectrogram patterns of individual fish scales can be adjusted to a common period accounting for differences in date of return or size of fish at return without biasing the growth profile of the scale. We apply the method to 147 Atlantic salmon scale images sampled from 3 years and contrast the information derived with this automated approach to what is obtained using classical human operator measurements. The spectrogram analysis quantifies growth patterns using the entire scale image rather than just a single transect and provides the possibility of more robustly analyzing individual scale growth patterns. This semi-automated approach that removes essentially all the human operator interventions provides an opportunity to process large datasets of fish scale images and combined with advanced analyses such as deep learning methods could lead to a greater understanding of salmon marine migration patterns and responses to variations in ecosystem conditions.

## Introduction

**Figure 1.**
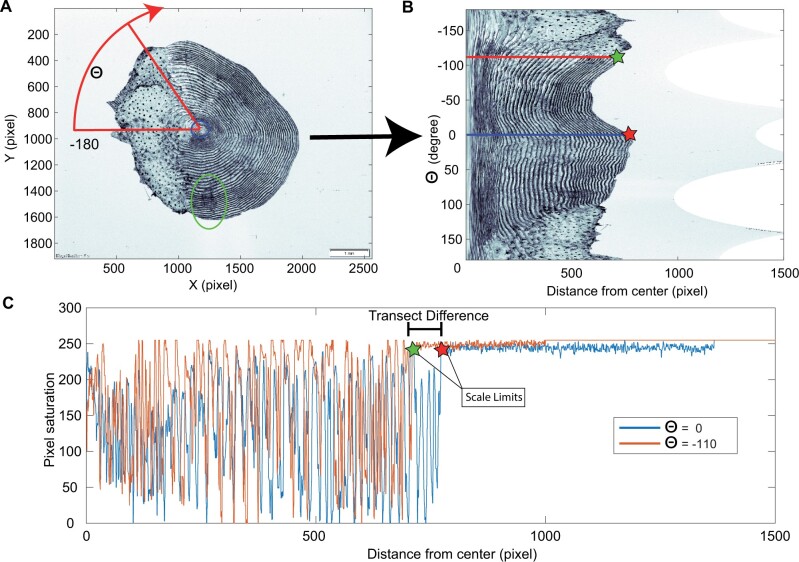
A) Example Atlantic salmon scale image from 2015. Each scale image is radially sampled according to angle θ, resulting in the unwrapped image in B). The operator placement of the focus within the center of the scale is depicted by the start of the red radial transect within the blue circle, and the green ellipse shows an example of a localized scale imperfection in panel A at angle θ = 90. C) Examples of two-pixel saturation vs radial distance transects for angles θ = 0 and θ = −110, showing the impact of anisotropy (non-circularity) of the scale on the extracted time series and the inherent problem of averaging inter-circuli distances from multiple transects.

The growth of many biological organisms varies seasonally and annually in response to variations in energy inputs, temperature, and age. These variations in growth rates are often recorded as distinct bands of cellular accumulation and aging (as in the case of trees [1]), as distinct patterns of mineralization of skeletal features (teeth of mammals [2]), or mineral depositions on dermal structures (as in fish scales [3]). Fish scales are mineralized layers of collagen, calcium, other minerals, and proteins that are formed from secretions of bone cells organized into individual scale pockets [3]. In bony fishes, there is a fixed number of scales on the body and the individual scale grows with the fish. In Atlantic salmon (*Salmo salar* Linneaus), the secretion of minerals is relatively continuous but seemingly at variable rates that appear as a sequence of stops and starts. The expansion period is characterized by a thin relatively transparent mineralized layer interspersed with accumulations of minerals (referred to as the stop portion) that form ridges on the scale called circuli and appear as dark lines on the scale image (Fig. 1A). The portions on the scale with wider inter-circuli spacings correspond to periods of more rapid growth in body size and scale pocket, whereas the portions with narrowed inter-circuli spacings correspond to periods of slower growth [4]. The sequence of wide (typically summer) followed by narrow (typically winter) inter-circuli spacings is used to distinguish growth seasons with one summer–winter cycle corresponding to a year of growth. This interpretation of the sea age for Atlantic salmon based on scale patterns has been validated by marking fish as they go to sea and analyzing their scale patterns when they return as mature adults after one, two, or more years at sea [5]. Delineation of circuli and measurements of inter-circuli spacing offer some insight into a fish's age, growth rate, and migratory status [3, 6, 7].

Atlantic salmon age and life history are most often interpreted from scales because they do not require sacrificing the fish, they are relatively transparent and thus require minimal pre-treatment prior to imaging, and for the fish, lost scales can quickly grow back although the subsequent growth history is lost on the regenerated scale. Scales sampled from fish returning to their natal rivers have been analyzed using various metrics to infer the size of the fish at different periods of freshwater and marine life for the purpose of characterizing differences in growth patterns commensurate with varying conditions experienced by the fish during their time in rivers and at sea [8, 9, 10, 11, 12, 13, 14]. Variations in growth rate indices among seaward migrating cohorts are then often compared to environmental and biological components of the marine ecosystem for example for the purpose of elucidating the factors that could be impacting fish size at a given age, marine survival rates, and other aspects of their ecology (see references above for example).

Although information rich, fish scales are imperfect recording instruments with noisy transfer functions. By analogy, each fish scale is a chart recording instrument with a drum diameter corresponding to the body size of the returning fish and the speed of rotation of the drum corresponding to the time spent at sea. The trace registered on the rotating drum or the scale is additionally modified by individual variations in metabolic rates and in rates of mineral excretion and deposition in the individual scale pocket. The shape and circuli patterns on Atlantic salmon scales also vary with the position on the body of the fish, and proximate scales from the same location can vary in shape and size [15]. As a result, a standardized scale sampling location has been defined in order to reduce the variability in scale shape and size relative to body size of salmon [7]. In practice, the scales collected from the fish by the sampler will vary somewhat within the sampling location and this sampling variability adds variance in the growth characteristics that are quantified from the sampled scale [16].

Currently, information extracted from salmon fish scales is obtained from digital images analyzed using software that allows the measurement of circuli positions and distances along a radial line defined by a human analyst [13]. The process of manual scale measurements, even for a single radial line, is time-consuming and is subject to substantial variation due to local imperfections in individual scales, known anisotropy (non-circular form) of scales and variations in individual analyst interpretations of scale shape and placement of the radial line [17]. As a result, studies using fish scales to quantify growth have mostly been limited to small datasets of a few thousand samples and largely focus on identifying periods of slow (winter) and rapid (summer) growth during the marine phase of salmon. A limited number of size metrics of different portions of the scale are extracted from the distance metrics along the single radial line; however, the bulk of the rich information present in the digital image of the scale is ignored [9, 10, 11, 12, 13]. Given somewhat random local perturbations and imperfections in ridge formation in scales [4, 16] (e.g. Fig. 1) and the errors introduced by manual scale interpretation and measurements [16, 17], there is a compelling need for a rapid and repeatable automated approach that reduces the need for human operator interventions for analyzing growth rates from fish scales.

The high-resolution digitization of fish scales provides opportunities for novel approaches for extracting information and characterizing growth patterns from salmon scales. With continuing progress in learning methods that not only replicate human behaviors but also uncover high dimensional information in large data sets, it is imperative that we develop automated methods for processing scale images and identify data transformations that not only highlight salient information while suppressing noise, but also reduce the dimensionality of the resulting data.

Here, we propose a novel semi-automated method based on Fourier spectral methods to analyze the ensemble average of growth pattern information extracted from digital images of fish scales. We illustrate the method and the approach using Atlantic salmon scales. The approach uses the individual pixel saturations along a series of radial lines over the entire fish scale image to characterize the growth profiles registered on the scale. Given the distinct pattern of peaks and troughs of pixel saturations associated with the circuli depositions observed in radial transects of the Atlantic salmon fish scale, a short window Fourier transform (SWFT) that provides a time/frequency description of that transect (called a spectrogram) yields a proxy (i.e. frequency) for circuli spacings as a function of distance from the center of the scale. The nearly circular nature of the scale growth process presents opportunities to extract consistent growth profiles and to generate ensemble averages that blunt the effect of local imperfections in scale patterns and human operator placements of transects and may lead to more robust and repeatable depictions of individual scale growth patterns. The automated quantification of the growth profile in terms of frequencies and averaging of these over the entire scale profile provides opportunities for extracting informative data from the entire scale that otherwise is not provided from a single-analyst-defined radial line. Resulting growth profiles could be used to explore links between environmental and ecosystem forcing and fish growth responses as recorded by sampled fish across years. Ultimately, automated methods could provide opportunities for extracting much more information from large databases of fish scale images that could be used to model ecosystem and climate effects on seasonal and annual growth trajectories.

## Methods and rationale

An automated method to analyze fish scales must be able to solve the following issues:

1. Scales often have local imperfections that frustrate automated circuli and annuli spacing estimates or require *ad hoc* rules for defining circuli [7, 18].
2. Scales are not perfectly circular, and thus directly averaging absolute distances of scale features over a variety of angular transects or averaging over replicate scales from the same fish is not possible because the distance from the center to the edge of the scale varies, despite the same total absolute growth time for an individual fish.
3. The posterior exposed portion of the scale does not feature circuli and annuli patterns.

We developed a semi-automated method to analyze pixel saturations along an ensemble of radial lines of an entire scale. For purposes of demonstrating this novel approach, we analyzed a dataset of 148 digitized Atlantic salmon scale images, approximately 50 per year sampled in 2005, 2010, and 2015. The scale samples were obtained from salmon that spent just over one year at sea (termed one-sea-winter salmon or 1SW) and returned to the Miramichi River (Canada) to spawn. The salmon were captured at the Fisheries and Oceans Canada Science Branch estuary trapnet and were released back to the wild after measuring for length and collecting a scale sample. The fork length of these fish ranged from 49.0 cm to 62.3 cm with mean fork lengths of 57.1, 54.3, and 54.2 cm in 2005, 2010, and 2015, respectively. These salmon had migrated to the ocean in the previous year; the precise date of migration is unknown for the individual fish but in the sampled river the juvenile salmon go to sea during a narrow window of mid-May to early June. The dates when the salmon returned to the river as mature adult fish and were scale sampled ranged from 8 June to 31 July, with mean dates of 18 July, 5 July, and 18 July in 2005, 2010, and 2015, respectively. In the laboratory, the scales were pre-washed of debris, dried, and those with complete growth profiles (i.e. small focal area where the scale grows initially after hatching) and no erosion of the scale edges were mounted on acetate or glass slides and photographed at an image resolution of 2560 X 1920 pixels, with a magnification equivalence of 302 pixels per mm. The scale was generally orientated horizontally relative to the longest axis of the scale. The chosen magnification provided a digitized image of the complete scale.

Each scale image was processed in a semi-automated fashion, with the user prompted to indicate via mouse click the location of the center of the scale focus for each sequential scale image; a specific pixel pair defines the starting point for all radial transects. Pixel saturation values, ranging between 0 and 255 in all images, were extracted from the image file along radial lines drawn at 0.1-degree intervals for a total of 3600 vectors (Fig. 1B). The dense sampling of the angle ensures that the outermost circuli are adequately represented in this “upwrapping.” Once this is accomplished (Fig. 1B), superfluous information such as the scale bar in the lower right corner of the image was automatically removed using an algorithm that detected the edge of the scale using a sliding window analysis of the pixel saturation that represented the falloff from the pixel saturations of the scale to the background luminance (for example, consistent low variance around an expected pixel saturation value for each image, though this would be adapted to a given dataset's image background color) (Fig. 1B, 1C).

**Figure 2.**
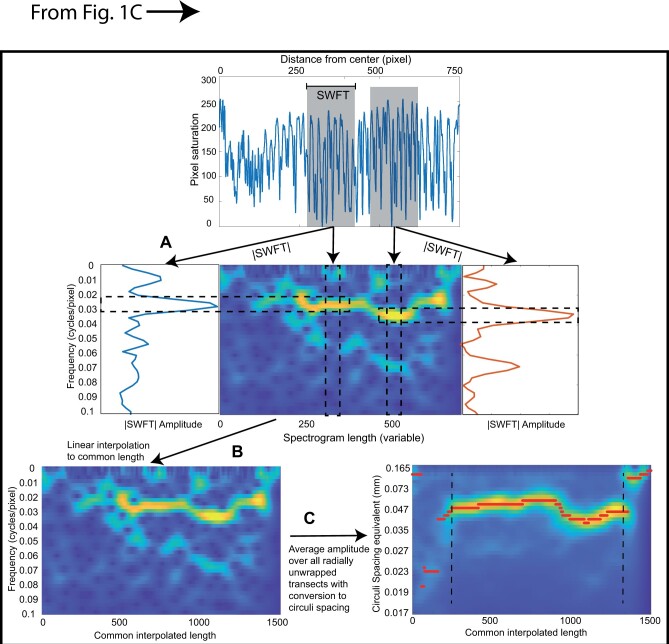
Description of transect spectrogram processing: A) SWFT are performed on a sliding window along a given angular scale transect. Each amplitude spectrum is inserted as a column of a spectrogram matrix (center color panel), where warmer colors represent higher amplitudes (i.e. blue is low, yellow is high). B) Interpolated spectrogram from panel A stretched to a common length of 1500 units. C) Ensemble average of the stretched spectrograms from the processed transects of one scale with the frequencies converted to circuli spacing equivalents. The maximum amplitude (red symbols) in the averaged spectrogram identifies the dominant frequency by position (from focus to the edge) for each scale.

The distinct pattern of peaks and troughs of the pixel saturations observed in the radial transects of the scale can be seen as a waveform, with the troughs in saturation values corresponding to the circuli locations (Fig. 1C). The Fourier transform decomposes any waveform into a sum of sinusoidals, a sequence of sine and cosine functions of varying frequencies. A sliding SWFT was used to provide a distance/frequency description of radially sampled transects, called a spectrogram (Fig. 2A). The frequencies quantified in the spectrogram are a proxy for circuli spacings found within the sampled window. The near monochromatic nature of a given transect's time series makes the extraction of the growth profile and suppression of the noise from the spectrogram a simple matter. This approach provides several advantages over operator-based methods, as described below.

We begin by defining a discrete Fourier transform over a small N-point window along the scale transect:

$$X\left(k\right)=\;\sum_{n=0}^{N-1}x\left(n\right)e^{-i\frac{2\pi k}{N}n}$$

where *N* is the number of points in the window, here chosen to be 200 based on the desired frequency resolution for this study (out of a total of 500–800 pixels per transect), *x(n)* is the raw scale transect sampled in the limited window (in one row of Fig. 1B), and *X(k)* is the Fourier transform of *x(n)*. The absolute value of *X(k)* is the amplitude spectrum (side panels of Fig. 2A).

Under a spectrogram mapping (Fig. 2A), the entire growth profile of a given transect is captured in terms of pixel position, frequency, and amplitude. SWFT are performed on a sliding window for each angular scale transect (Fig. 2). The SWFT of each window results in a Fourier transform, the absolute value of which is the amplitude spectrum with units of amplitude vs frequency (cycles/pixel); examples from two windows are shown on either side of the center panel in Fig. 2A. To avoid wrap-around artifacts inherent in discrete Fourier transforms, each window is multiplied by a Hamming taper to ensure that the left and right sides of the window taper off to 0. Here, Fourier transforms and tapering windows were obtained using the “fft” and “Hamming” functions in Matlab©, but such functions are broadly available in other languages as well.

Several parameters are adjusted to calculate the spectrogram of a transect (Fig. 2A), namely window length and increment step size. In this study, we chose a window of 200 pixels which is moved forward in five-pixel increments from the focus identified by the operator and the process is repeated up to the automatically flagged end of the scale (Fig. 1C, red and green stars). Shorter sliding window lengths will compute the amplitude spectrum based on a smaller number of pixel values but for the same total frequency range, thus reducing the frequency resolution (i.e. the spacing between each frequency on the x-axis) but it will increase the spectrogram's time resolution (i.e. how rapidly transitions in spectrum behavior will be observed in the spectrogram). A smaller increment step results in a larger and smoother spectrogram matrix at the cost of computational power and may not be necessary to resolve meaningful growth rate information.

The amplitude spectrum from each window (Fig. 2A) is sequentially inserted as a column of a spectrogram matrix (Fig. 2A, center color panel), converting the individual window amplitude spectrum (with dimensions frequency and amplitude) into a three-dimensional representation with dimensions x = window position along the transect, y = frequency, and z = amplitude; amplitude values are colored in Fig. 2 with warmer colors representing higher amplitudes (i.e. blue is low, yellow is high).

An ensemble average of the growth profile of each scale is derived from all the radial line spectrograms. However, as each transect is of different length (e.g. Fig. 1B), the resulting spectrograms have different x-dimensions and averaging over multiple radial lines requires an absolute time or length scale (i.e. each radial line must have the same time or distance axis along the scale in order to be averaged). Directly stretching and interpolating a given radial line pixel saturation values to a common length prior to spectrogram analysis would effectively bias the circuli spacing information and the mean over all transects for the scale. It can be observed, however, that for a given period (e.g. ocean growth), circuli spacing sequences are similar across angular transects of the scale and the variations in transect length and circuli numbers and positions are a consequence of the discrete creation/destruction of circuli [18] (Fig. 1B).

To average over all spectrograms, we thus resample the spectrogram from each transect to a common length in the x-direction using linear interpolation, which is equivalent to sampling the unstretched spectrogram at a higher sampling intensity. This common length can be chosen as anything as long as it is longer than any transect sampled over the entire data set to avoid data loss. For each frequency *y* at any two known points ($x_{0}^{\prime }$*,* $z_{0}$) and ($x_{1}^{\prime }$*,* $z_{1}$) where *x′* (*x/xmax*) is the rescaled [0,1] position on the x-axis of the spectogram of transect length *xmax* and *z* is the amplitude at *x'* of the spectrogram, any interpolated point $z^{\prime \prime }$ for a desired stretched scaled position *x''* in the interval [0,1] is calculated as:

$$z^{\prime \prime }=\;z_{0}+\;\left(x^{\prime \prime }-x_{0}^{\prime }\right)*\;\left[\left(z_{1}-z_{0}\right)/\left(x_{1}^{\prime }-x_{0}^{\prime }\right)\right]$$

The frequency content of the spectrogram is unchanged in this interpolation, but its x-dimension is increased to an arbitrary common value (here chosen to be 1500) (Fig. 2B). This would be adjusted based on the pixel size of the transects from all the processed images in the study. After stretching the angular transect spectograms to a common length, the ensemble average of the spectrograms of all the angular transects is calculated. Unlike directly stretching the scale transects in Fig. 1C, stretching the spectrogram (Fig. 2B) preserves the spectrogram profile, and thus a subsequent average over all *θ*-transects results in a more complete growth pattern for that scale with significant reductions in noise (Fig. 2C). Imperfections and areas of the scale that do not feature circuli information contribute weakly and randomly to the ensemble average because there are less distinct and dominating frequencies and lower amplitude values.

**Figure 3.**
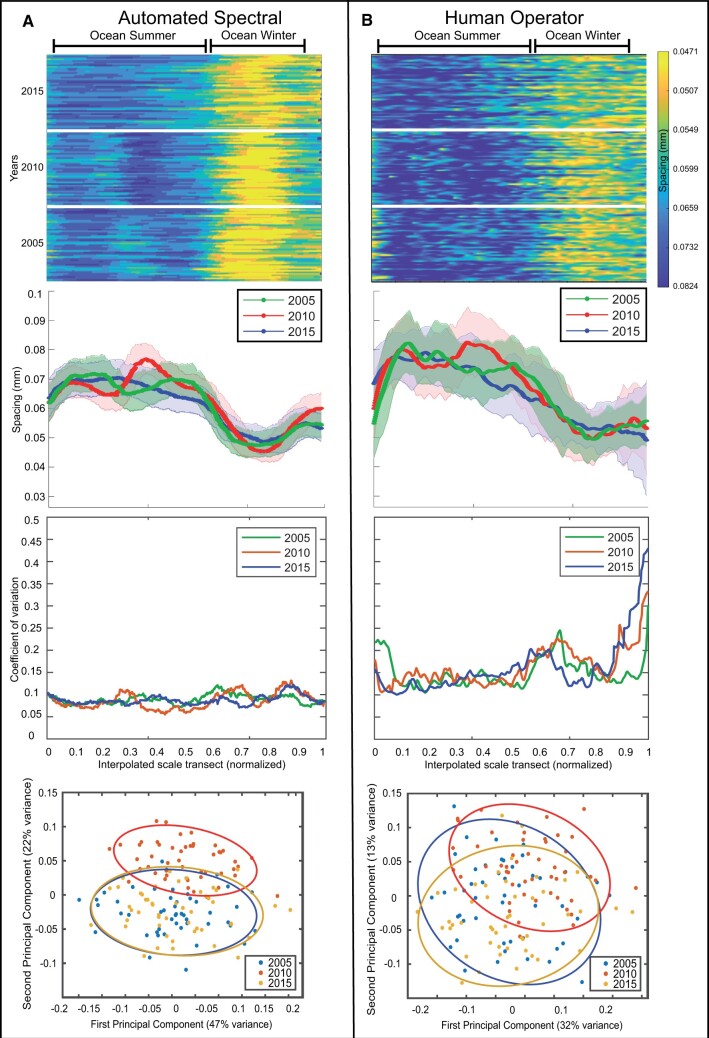
A) Automated spectral measurements and B) Human operator measurements. From top to bottom: 1) Primary spectral trend from averaged spectrograms (Fig. 2D) for each scale compared with manual measurements of circuli spacing, by year and sorted by sampling date from earliest to latest in each year. 2) Mean (and 1 standard deviation polygon) growth pattern for each year by position in the first year of ocean residence. 3) Coefficient of variation by position in the first year of ocean residence. 4) PCA scatter plots of samples and 95% confidence ellipses by year for the first two principal components.

From the ensemble average spectrogram, the frequency with the maximum amplitude by stretched position is retained for each scale (the red line highlighted points in Fig. 2C). Extracting the dominant and largely monochromatic trend from the spectrogram is a matter of tracing the maximum peaks and this yields a vector of frequencies by stretched pixel position for each scale that can easily be compared among samples (Fig. 3A). Although the default unit of the amplitude spectrum is cycles/pixel, this can be converted back to units of circuli spacing in millimeters using the given image magnification of 302 pixels per millimeter. Note that the inter-circuli spacings unit is the reciprocal of frequency with high frequencies translated into narrower inter-circuli spacing equivalents.

In the interest of examining the growth patterns of salmon during the first year of ocean residency, the freshwater portions of the scale defined by very narrow spaced circuli (or poorly defined or high frequencies) adjacent to the focus and the narrower spaced circuli representing the phase of return to rivers at the tail end of the scale (black dotted lines, Fig. 2C) were identified and removed using a sliding window variance analysis. This was done computationally by looking at the variance of the peak maxima for each column of the ensemble of the stretched image (Fig. 2C). The fresh water and the return to rivers periods tend to feature poorly resolved circuli in images due to their close spacing; as such, peak frequencies resolved during these times tend to have broader distributions. In contrast, the first year of ocean growth represented by widely spaced summer circuli and narrowly spaced winter circuli result in consistently well-resolved circuli patterns and the vast majority of radial transects report the same maximum frequency with low variance. We thus search from left to right, and subsequently from right to left, in the spectrogram images and seek prolonged sequences of low peak variance (Fig. 2C). In this study, the start and end of the first-year marine growth phase were defined as the first (for the start) and the last (for the end) of a sequence of 30 consecutive low variance peaks (i.e. $\sigma^{2}$ < 10).

For comparison, we also present the classic scale metrics of inter-circuli spacings collected along one radial line of the scale as used in the majority of Atlantic salmon studies. Circuli positions and inter-circuli spacings along a single standardized user-defined transect were collected using ImagePro© software and provided by C. Breau (pers. comm. Fisheries and Oceans Canada, Moncton, NB, CANADA). This conventional sampling of the scale results in a nonlinear x-axis with the position of these measurements along an x-axis sampled at uneven points (e.g. 2-4-7-12-14-16 mm as cumulative distances along the transect corresponding to circuli numbers 1 to 6). The inter-circuli spacings are a quantification of the rate of growth of the scale, per circuli deposited. As with the spectrogram interpolations of frequencies, rate of scale growth equivalents for a standard number of sampling points along a standard transect length (on the interval 0 to 1 based on maximum length of each transect) were calculated by linear interpolation using inter-circuli spacing values at adjacent rescaled cumulative distances along the transect. For equivalence to the stretched spectrogram approach, a total of 1500 sampled values of inter-circuli distances were interpolated along the rescaled (0 to 1) transect length. For comparative purposes and for correspondence to the period of growth for the first year in the ocean shown for the spectral method, the stretched inter-circuli spacings plots were visually truncated at a common stretched transect point that approximately corresponded to the end of the ocean winter period (Fig. 3B).

As a basic demonstration of raw information content gained by the spectrogram mapping of the scale compared to the classic method of inter-circuli spacings from one transect, we perform a principal component analysis (PCA) decomposition of the interpolated inter-circuli spacing patterns for the first year of ocean growth along the standardized transect length for each individual fish. The distributions and 95% multivariate normal confidence ellipses of the individual samples by year are shown for bivariate scatter plots of the first two PCA components for each method. The PCA analyses were done with the “pca” function in Matlab©.

## Results

The human-operator-extracted total number of marine circuli along a single transect of the individual fish scale are variable within and among years. Scales sampled from fish in 2015 show fewer marine circuli (mean = 42.6, range 33 to 52) than those of 2010 (mean = 45.2, range 38 to 55), and 2005 (mean = 48.2, range 40 to 58), consistent with smaller mean lengths of fish sampled in 2015. The scale growth profiles by analysis method for the first year of ocean growth of individual sampled fish are shown in the top panels of Fig. 3, organized chronologically in terms of fish sampling date within year. For the spectral method, the common-length vector of the spectral frequency representing the growth pattern of each processed scale was converted from frequency (cycles/pixel) to circuli spacing equivalents (mm). The automated spectral patterns have some offsets (undefined frequencies at the trailing edges for some samples) due in part to the varying sizes and dates of sampling of the fish, metabolic rates differences, and algorithmic variations in defining the ocean start and end dates. The distance-adjusted human operator measurements are shown in the top panel of Fig. 3B. Identical color scales for the spacings are used for the two methods.

Ocean summer and winter periods are easily identified as the respective broad and mostly blue versus narrow yellow bands in Fig. 3, top row panels. For the spectral method, the intra-year growth patterns are visually strongly correlated in terms of modulation for the sampled years 2005 and 2010. Averaging over a large number of scale transects thus allows long period modulation patterns affecting all fish in a given year to emerge strongly (Fig. 3A top and middle rows), rather than somewhat weakly as in the ensemble average of classical circuli spacing patterns of a given year (Fig. 3B).

The 3 years used in this article to demonstrate the automated technique were selected *ad hoc* from the available image database, and the growth profile patterns are quite striking in the differences among the 3 years. For the Fourier automated approach, the summer ocean growth patterns are described as follows:

- In 2005, a moderate period of fast growth (blue) was followed by a moderate period of slow growth (teal) and another period of fast growth (blue) before the winter.
- In 2010, growth was defined by an extended period of mostly slow growth (blue-teal) followed by a short period of very rapid growth (deep blue color) leading into winter. Notably, the pattern of slow/fast is offset in time from 2005 to 2010, and is more severe in 2010.
- In 2015, somewhat heterogeneous moderate and rapid growth within and among fish is observed throughout the summer that slowly tapers off toward winter.

The coefficients of variation for the Fourier automated approach are substantially smaller than for the human-operator-derived growth metrics (two middle rows of Fig. 3). There is also strong inter-year variation pointing to potentially variable global forcing effects on the growth of fish populations in a given year. These effects are significantly more obscured in human measurements (Fig. 3B). The first two PCA components in the Fourier approach explain almost 70% of the total variance in the automated spectral patterns, compared to 45% for the human operator method. The spectral method pattern of scales in 2010 is prominently separated from the other two years although strong overlap in the first two components is still observed for 2005 and 2015 (Fig. 3 bottom row).

## Discussion

Beyond the obvious advantage of removing essentially all human operator inputs and automating the circuli position and spacing measurements, the approach described here provides an opportunity to use the growth patterns registered on the entire scale image rather than from a single variably positioned transect. Where single transect data are prone to variations due to scale imperfections, human imprecision in placement of the transect line and identification of circuli, and the inherent noisy local response of the scale pattern to growth of the fish [18], averaging over the entire image suppresses much of the variation and promotes the emergence of more robust underlying growth patterns. This is clearly apparent in the side by side comparison in Fig. 3, where more homogeneous growth patterns, particularly in years 2005 and 2010, emerge from the automated spectral analysis, but these are obscured or absent in the human operator derived patterns. This result has several methodological and interpretive implications, as discussed below.

## Considerations for flexible generalization of the method

The method described in this article was developed for a specific image set, but every step in the approach was automated after the user input of the center focus for each image (which could also be automated in the future). The automation of the extraction of data from a scale image opens the door to analyzing large numbers of scale images, and to processing multiple scales from the same fish to address some of the concerns regarding the representativeness of growth profiles of individual fish extracted from a single scale and/or a single transect from the scale [16]. The translation of pixel saturations into frequencies of a spectrogram which can then be stretched to a common time/length for each extracted transect of data resolves the problem with averaging growth information based on absolute distances due to the characteristic anisotropic form of salmon scales. Finally, by extension, stretched spectrogram profiles could be averaged for replicate scales from individual fish, thus resolving the problem of incorporating inter-scale variation in the characterization of growth patterns of individual fish.

A few parameter choices in the described method can impact the way images are processed. Image resolution is an important consideration in defining the spectrum window and the step size to estimate a scale transect spectrogram. The digital images analyzed here are of medium resolution (2560 X 1920 pixels) and the magnification used provided an image of the entire scale within the image frame. If the resolution is too coarse (for ex. the image resolution in the study of Vabo et al. [19] of 380 × 380 pixels), the circuli and inter-circuli spacings would be characterized by a smaller number of pixels, and thus a smaller sampling window would need to be used. As a consequence, there would be less information (pixels) per cycle to define the spectrogram, and the frequency resolution would be diminished. The high-resolution images (4800 × 3600 pixels) used by Tillotson et al. [14] would likely have been excellent data to analyze the spectral patterns of circuli but a longer sampling window would have been needed to define the spectrogram. Note that image resolution should not affect the estimates of the dominant frequency signatures of the scale, only the spacing between different frequencies in the Fourier transform (i.e. spectrum resolution in Fig. 2B). Also, since it is the relative pixel saturation range that is used to define the circuli depositions and cycles, variations in image brightness among scales should not be of concern. Cleanliness of the scale is however important as dirt or other non-circuli-related debris would appear as variations in pixel saturations and could therefore bias the analyses. Provided the dirt and other imperfections are minor, these would likely be averaged out, much like natural imperfections of the circuli patterns, using a large number of transects from the entire scale image.

The longest axis, from focal center of the scale to the edge, included approximately 600 to just under 1000 data points (pixels of saturation), with fewer data points for the radii along the edges of the scale (see Fig. 1 for example). A window of 200 pixels was used to estimate the amplitude spectrum and this window was moved by five pixels to the edge of the scale. For the salmon scales analyzed, the 200-pixel window would comprise approximately 10 or more cycles of pixel saturations for the longest transects and allow for a spectral resolution of approximately 0.05 cycles/pixel. The number of cycles within a transect would be less for the shorter transects along the edges of the scales as would the number of windows applied to the transects. Given these tradeoffs, it is always recommended to adapt the spectrogram window length to the resolution of the data.

Although we use a sliding window to describe the spectral pattern of the transects, this should not be conflated with a smoothing of the pattern per se. Each Fourier transform simply returns the various amplitudes of sinusoidal components in the chosen window (e.g. Fig 2A). Since we are only retaining the largest frequency amplitude (i.e. the peak in Fig. 2C), then sharp transitions in spectral behavior will still be recovered. The key to robustly estimating the frequency patterns of the circuli is including a sufficient number of cycles, or circuli and inter-circuli patterns, in the chosen window. A very short window may correctly isolate a single frequency of interest, but may also be vulnerable to noise and data artifacts, and feature very poor actual spectral resolution. A longer window conversely will feature high spectral resolution, but will not be necessarily representative of the center point of the window to which the Fourier spectrum is mapped in the spectrogram.

Other adaptations can also be made to our approach. For instance, users could be provided with the option to set, post hoc, limits on the angular range in Fig. 1B to be retained for analysis, potentially lowering the variance in the spectral average and allowing the study of the fresh water growth phase, for example, where, for the most part circuli are not well resolved by the available image resolution.

The samples processed in this study are limited to salmon which spent one year at sea; however, the method is inherently generalizable to fish of other sea ages, with some considerations. There is a clear and evident transition between fast and slow growth periods in the ocean, and these would be recovered as a series of progressively closer spaced cycles in multi-year fish. The stretching process of the spectrogram among sea ages could use body size or an average time spent at sea for the different sea ages as a standardization metric such that the growth patterns of single and multi-year fish could be aligned to match the corresponding period of time in the ocean, such as the first year of growth in multi-year fish. This could be automated via iterative stacking algorithms that would match patterns in single-year fish (for example the frequency pattern from the winter slow growth) with stretched portions of those seen in multi-year fish. Differences in magnification to capture the entire scale image for fish of different sea ages (the bigger the fish, the larger the scale) would have to be accounted for as this would affect the sliding window and step size. If the scale images of some fish are partially cut off under a chosen magnification, then restricting the analyses for all scale images to transects within a given radial range could be an option.

## Interpretation of growth modulations

Growth of the scale and that of the fish are not linear in time. The growth rate in length and the rate of circuli deposition (days between successive circuli) depend on temperature and feeding rates [4, 20] with fewer days between circuli at fast growth rates and more days when growth rates are slower [21]. In this example analysis, we focused on the spectral pattern corresponding to our interpretation of the first year of ocean residency. There is much debate regarding the dates within the year when specifically the period of slow growth, interpreted as the winter, is registered on scales. Some authors proposed or assumed that growth rates are correlated with photoperiod with the midpoint of the zone of closer inter-circuli spacings registered at the winter solstice [22, 23, 24]. More recently, the zone of narrowly spaced circuli was estimated to be deposited during a period of decreasing ocean temperatures in mid-February to late March likely encountered by salmon in the Labrador Sea and the subsequent wider-spaced circuli would be deposited when sea surface temperatures would be increasing in the spring, consistent with ecosystem conditions, bioenergetics, and biological responses of salmon [25].

The scale growth patterns resolved with the spectral method shown in Fig. 3A display robust and unique intra-year similarities during summer ocean growth periods suggesting that fish growth and the corresponding growth patterns may most strongly be influenced by broad oceanic conditions (temperature and food) affecting all the fish during the same migration year.

## Increase in information content

Classic absolute metrics of scale increment size based on inter-circuli distances, post-smolt growth increments, and back-calculated size of fish from scale size have been extensively used to study the associations between variations in average size of fish and survival at sea [9, 10, 11, 12, 13]. There is a substantial amount of growth profile information registered on scales that is ignored when only the absolute metrics of size along a single transect are used. The advantage of the approach described in this article from the analysis presented in the references cited above is that the spectrogram analysis has been automated, uses information from the entire scale image, and characterizes the growth profiles using the spectrogram patterns that can be adjusted for differences in date of return or size of fish at return without biasing the growth pattern of the scale. The stretched spectrogram pattern derived from averaging spectrogram frequencies rather than absolute distances from a large number of transects from the entire scale image addresses the problems related to the anisotropic characteristics of scales and characterizing growth patterns from different areas of the scale [18] as well as from more than one scale from the same fish.

The spectral method pattern in 2010 in particular is much more prominently separated from the other two years than for the human-operator-derived metrics, although there remained a strong overlap in distribution of the samples from 2005 and 2015 based on the first two PCA components (Fig. 3 bottom row). This may be due to the PCA being a linear method, and thus inherently limited in how it is able to translate the variance characteristics of these data. The growth profiles resolved from the spectral analysis of a large dataset of scales would be amenable to exploration using alternative multivariate time series approaches such as dynamic factor analysis and nonlinear basis pursuit methods (e.g. neural network autoencoders, independent component analysis, etc.) that have shown an impressive ability to overcome the limitations of the illustrative PCA analysis. A salient point of the described spectral method is thus one of data mapping, as is common in many disciplines, that promotes the important information in a dataset while suppressing noise prior to learning its features.

The analysis of large databases for pattern determinations is not a new idea, but leveraging machine learning for increasingly large data sets of this type is novel in this field. Advances in deep learning using convolutional neural network (CNN) classifiers have been applied directly to scale images that roughly rival human operator labeling of several scale-derived metrics, such as river and marine ages, fish origin (wild or farmed), and spawning history [19]. This approach effectively simulated human operator notations of scales, and as such suffered from the same limitations as human operators in terms of class objectives. Notably, more tightly spaced annuli and corresponding river ages were poorly constrained given that this task also features high variance among human operators (and thus is trained with inherently high variance). As a supervised learning approach trained on optimizing specific metrics, it is approximately capable of replicating human operator conclusions but cannot reveal new information unseen by human operators (such as unusual growth patterns, climate effects, or clustered growth behaviors). Furthermore, CNN-based architectures provide forms of approximate invariance (notably with respect to translation) that may be unsuited to scale images, since most of the salient information in a scale is approximately invariant with respect to rotation around the scale center, and thus other data representations are likely preferable. Pre-treatment of the scale images using the automated spectral method described here prior to inputting could improve the performance of convolutional neural networks in the interpretation of fish age, growth pattern features, and other classifications of interest.

## Conclusion

Although this is a demonstrative methods article framed in terms of classic studies of Atlantic salmon growth trajectories from scale patterns, the automated analysis of scale images described here could be used to refine the classic analyses, incorporate data from replicate scale samples from the same fish, and envision new analyses using fish scales. The automation of the extraction of data from a scale image opens the door to analyzing large numbers of scale images. The translation of pixel saturations into frequencies of a spectrogram which can then be stretched to a common time/length for each extracted transect of data solves the problem with averaging growth information based on absolute distances due to the characteristic anisotropic form of salmon scales. Finally, by extension, stretched spectrogram profiles could be averaged for replicate scales thus resolving the problem of incorporating inter-scale variation in the characterization of growth patterns of individual fish.

The old adage states: “you don't know what you don't know.” Seasonal and annual growth patterns are noted on structures in a diversity of biological organisms from trees [26], to fish (as described in this manuscript), to mammals [2]. In some example studies for tree ring analysis [26], measurements are done manually and along a single transect of a digitized image and we can see how the semi-automated spectral analysis described here could greatly enhance the within-year and among-year growth pattern information extracted from such images. In the case study and novel semi-automated method presented here, it is clear that many more samples could be rapidly processed and much more information that is classically used in analysis of growth patterns from fish, trees, and mammals, to name a few, could be obtained.

## Data Availability

The digitized Atlantic salmon scale images were obtained as part of a research project on responses of Atlantic salmon populations to changes in marine ecosystems led by Cindy Breau of Fisheries and Oceans Canada, Science Branch, Moncton (Canada). We are grateful to Cindy for providing us access to the three years of images processed in this manuscript and for providing the corresponding classic scale metrics and the biological metadata of the sampled scales. Scale images and classic scale measurement data can be requested by contacting C. Breau at Cindy.breau@dfo-mpo.gc.ca.

## Code availability

The source code for the described method is available from the primary author (J.A.C.) on request.
